# Transcriptional Regulation of 15-Lipoxygenase Expression by Histone H3 Lysine 4 Methylation/Demethylation

**DOI:** 10.1371/journal.pone.0052703

**Published:** 2012-12-28

**Authors:** Cheng Liu, Dawei Xu, Hongya Han, Yidong Fan, Frida Schain, Zhonghua Xu, Hans-Erik Claesson, Magnus Björkholm, Jan Sjöberg

**Affiliations:** 1 Department of Medicine, Division of Hematology, Karolinska University Hospital Solna and Karolinska Institutet, Stockholm, Sweden; 2 Department of Urology, Qilu Hospital, Shandong University, Jinan, People’s Republic of China; Texas A&M University, United States of America

## Abstract

15-Lipoxygenase-1 (15-LOX-1) oxidizes polyunsaturated fatty acids to a rich spectrum of biologically active metabolites and is implicated in physiological membrane remodelling, inflammation and apoptosis. Its deregulation is involved in the pathogenesis of diverse cancer and immune diseases. Recent experimental evidence reveals that dynamic histone methylation/demethylation mediated by histone methyltransferases and demethylases plays a critical role in regulation of chromatin remodelling and gene expression. In the present study, we compared the histone 3 lysine 4 (H3-K4) methylation status of the 15-LOX-1 promoter region of the two Hodgkin lymphoma (HL) cell lines L1236 and L428 with abundant and undetectable 15-LOX-1 expression, respectively. We identified a potential role of H3-K4 methylation in positive regulation of 15-LOX-1 transcription. Furthermore, we found that histone methyltransferase SMYD3 inhibition reduced 15-LOX-1 expression by decreasing promoter activity in L1236 cells. SMYD3 knock down in these cells abolished di−/trimethylation of H3-K4, attenuated the occupancy by the transactivator STAT6, and led to diminished histone H3 acetylation at the 15-LOX-1 promoter. In contrast, inhibition of SMCX, a JmjC-domain-containing H3-K4 tri-demethylase, upregulated 15-LOX-1 expression through induction of H3-K4 trimethylation, histone acetylation and STAT6 recruitment at the 15-LOX-1 promoter in L428 cells. In addition, we observed strong SMYD3 expression in the prostate cancer cell line LNCaP and its inhibition led to decreased 15-LOX-1 expression. Taken together, our data suggest that regulation of histone methylation/demethylation at the 15-LOX-1 promoter is important in 15-LOX-1 expression.

## Introduction

15-LOX-1 is a peroxidase which catalyzes the oxygenation of free or membrane-bound polyunsaturated fatty acids containing at least one bis-allylic methylene [1]. It is implicated in various physiological processes including membrane remodelling, cell differentiation, inflammation and apoptosis [2], [3]. Deregulation of 15-LOX-1 expression is suggested to be involved in the pathogenesis of diverse malignancies, including prostate and colorectal cancer [4], , asthma [6], [7], atherosclerosis [8], orbital fibrosis [9] and nephritis [10]. Moreover, introduction of 15-LOX-1 into cells could result in oxidative stress and membrane degradation [11], [12]. Therefore, the expression and activity of the enzyme are strictly controlled.

In most 15-LOX-1 inducible cell types, the enzyme is predominantly activated through the IL4/13-signal transducer and activator of transcription 6 (STAT6) cascade [13], [14], [15]. 15-LOX-1 mRNA transcription is also associated with CpG island methylation status and histone acetylation status at the promoter level [16]. Different experimental evidences suggest that histone acetylation is positively correlated with 15-LOX-1 transcriptional activation [13], [16], [17], [18], [19]. In a previous study of HL cell lines we showed that DNA hyper-methylation is associated with silenced 15-LOX-1 transcription and that demethylation is required for 15-LOX-1 transactivation [16]. However, it was recently reported that hypermethylation of specific CpG di-nucleotides in the 15-LOX-1 promoter leads to the upregulation of 15-LOX-1 expression and enzyme activity in prostate cancer cells [20]. Moreover, recent work on colorectal cancer showed that 15-LOX-1 promoter methylation levels did not significantly correlate with 15-LOX-1 mRNA expression levels in neither cancer cell lines nor in the patients’ tumor specimens [21]. Therefore, additional epigenetic mechanism(s) could be involved in the transcriptional regulation of 15-LOX-1, controlling the tissue- and cell-type specific 15-LOX-1 gene expression.

Lysine is the key substrate residue in histone methylation, which can occur one, two or three times (mono-, di- or trimethylation), leading to different biological outcomes. Histone methylation could have various effects on gene transcription, depending on the precise residues and levels of methylation [22]. Generally, histone 3 lysine 4 (H3-K4) tri- and di- methylation have an activating effect on gene expression [22]. Histone methylation status of specific residues is an outcome of a dynamic balance between corresponding histone methyltransferases (HMTs) and histone demethylases(HDMs) [23].

HMTs are histone-lysine/arginine N-methyltransferases that catalyze the transfer of methyl groups to lysine/arginine residue of histones. Among the HMTs, SET and MYND domain-containing protein 3 (SMYD3) is a HMT that contains a SET domain and has histone H3-K4 di- or tri-methyltransferase activity [24]. SMYD3 is also a transcription factor that specifically interacts with the binding motif/s of its downstream genes. Endogenous expression of SMYD3 is undetectable or very weak in most normal human tissues whereas significant up-regulation was observed in the great majority of investigated colorectal carcinoma, hepatocellular carcinoma, and breast cancer specimens [24], [25]. SMCX, also known as KDM5C or JARID1C, has H3-K4 tri-demethylase activity and reverses H3-K4 to di- and mono- but not unmethylated products, and thereby functions as a transcriptional repressor [26].

We have recently reported that 15-LOX-1 is expressed in the HL derived cell line L1236 and in the tumor cells, the so-called Hodgkin/Reed-Sternberg (H/RS) cells, in classical HL. However, another HL-derived cell line, L428, lacks detectable 15-LOX-1 expression and activity despite the expression of functional IL4 receptors and active STAT6 [17]. In the present study, we compared the H3-K4 methylation status of the 15-LOX-1 promoter region between the two cell lines and found a relationship between H3-K4 methylation status of the 15-LOX-1 promoter region and 15-LOX-1 gene expression. We also studied how the HMT SMYD3 and the HMD SMCX exert their regulatory effects on 15-LOX-1 transcription. In conclusion, evidence supporting a close correlation between promoter histone methylation/demethylation status and 15-LOX-1 gene transcription is presented.

## Experimental Procedures

### Cell Lines and Culture Conditions

The HL-derived cell lines L1236 and L428 (kind gifts from Professor V. Diehl, Department of Internal Medicine, University Hospital of Cologne, Germany) and the prostate cancer cell line LNCaP were cultured at 37°C in RPMI 1640 (Life Technologies, Paisley, Scotland) containing 10% fetal calf serum (FCS), 100 units/mL penicillin, and 2 mM L-glutamine. L1236 was derived from peripheral blood of a patient with mixed cellularity HL, and L428 from pleural effusion of a patient with nodular sclerosis HL. Both cell lines are of B cell phenotype and negative for Epstein–Barr virus [27]. LNCaP were purchased from the American Type Culture Collection (Manassas, VA).

### RNA Extraction, Reverse Transcription and Real-time PCR (QT-PCR)

Total cellular RNA was extracted using the RNeasy total RNA isolation kit (Qiagen GmbH, Hilden, Germany) according to the manufacturer’s protocol. cDNA was synthesized using random primers (N6) (Pharmacia, Uppsala, Sweden) and M-MLV reverse transcriptase as described before [28]. Real time PCR with 3 µl of cDNA was performed in an ABI 7700 sequence detector (Applied Biosystems, Foster City, CA, USA) using Pre-made Gene Expression Assays (Applied Biosystems) primers and probes for 15-LOX-1 (Probe ID: Hs00609608_m1). Levels of transcripts were expressed as the ratio versus human beta-2 microglobin (Probe ID: Hs00187842_m1).

### Western Blots

Total cellular proteins were extracted with M-PER Mammalian Protein Extraction Reagent (Pierce, IL) according to the manufacturer’s instruction, and 10 mg of the protein were resolved by 4–15% SDS-PAGE (Bio-Rad, CA, USA) and transferred to a PVDF membrane. The membrane was probed with antibodies against 15-LOX-1 (made in-house by using purified recombinant human 15-LOX-1 as immunogen [29], SMCX (Bethyl Laboratories, TX), SMYD3 (Abcam, Cambridge, UK) or β-actin (Santa Cruz Biotechnology, Santa Cruz, CA) followed by anti-rabbit or goat horseradish peroxidase–conjugated IgG and developed with the enhanced chemiluminescent method (GE Healthcare, UK).

### Reporter Vector Construction

Genomic DNA from L1236 cells was purified using the Wizard Genomic DNA Purification Kit (Promega, Madison, WI, USA). A 1085 bp fragment of the 15-LOX-1 promoter region (NCBI sequence code: NT_010718) was obtained by high fidelity PCR (Roche, Switzerland) using primers binding to −1085 and −5 relative to the ATG codon. This fragment was ligated into pGL3-basic and named as pGL3-15-LOX-1 wild type (WT) (Promega). The cloned fragment was sequenced and showed the normal cytosine at position −292 [30].

### Luciferase Activity Assay

Cells cultured in 24 wells plates were cotransfected with pGL3-15-LOX-1 WT and pcDNA-SMYD3/pcDNA (a kind gift by Drs. Nakamura and Furukawa, University of Tokyo) or SMYD3 siRNA/non-specific siRNA using LipofectAMINE 2000. A Renilla luciferase-containing plasmid, which is driven by the thymidine kinase promoter, was always included in the transfections to control for transfection efficiency. Luciferase activity was determined by using a dual luciferase reporter assay system (Promega) 60 h after transfection. The 15-LOX-1 promoter-driven luciferase activity was normalized to the thymidine kinase Renilla activity.

### siRNA Treatment

Chemically modified Stealth siRNA targeting SMYD3 (Oligo ID: HSS127693), SMCX (Oligo ID: HSS112079) and control siRNA (Stealth™ RNAi Negative Control) were purchased from Invitrogen (Carlsbad, CA, USA). siRNA transfection was performed using LipofectAMINE 2000 according to the manufacturer’s protocol.

### Chromatin Immunoprecipitation Assay (ChIP)

ChIP assay was performed as described before [31]. Briefly, cells were cross-linked by incubation in 1% (v/v) formaldehyde-containing medium for 10 min at 37°C and then sonicated to achieve soluble chromatin with DNA fragments between 200 and 1,000 bp. Antibodies against trimethylated H3-K4, acetylated histone H3 (Upstate, Lake Placid, NY, USA), STAT6 (Santa Cruz Biotechnology, Santa Cruz, CA, USA), SMYD3 (Santa Cruz Biotechnology), and SMCX (Bethyl Laboratories, TX), were used to precipitate DNA fragments bound by their corresponding elements. The protein-DNA complex was precipitated with protein A Sepharose beads (Upstate), eluted, and reverse cross-linked. Following a treatment with Protease K (Sigma), the samples were extracted with phenol-chloroform and precipitated with ethanol. The recovered DNA was re-suspended in TE buffer and used for quantitive-PCR amplification as described previously [17] with primer set A (forward: 5′- ATTCCAGGGTGACAGAGTGAGACT-3′ and reverse 5′- CAGTTTCTTTTTGGGCTGGA -3′), primer set B (forward: 5′- CCCACCTCGCCTGCCTGCT-3′ and reverse 5′- TTGGGGTCGGAATGTGGGTG-3′) or primer set C (forward: 5′- AATCCTTTTCTTTTTCTCC-3′ and reverse 5′- TAACCCAGCCT GAGACCC-3′).

### 15-Hydroxyeicosatetraenoic Acid (15-HETE) Assay

The capacity to produce 15-HETE was used as an indication of 15-LOX-1 activity in the living cells. The levels of 15-HETE were measured by an EIA Kit (Cayman, USA) according to the manufacturer’s protocol.

### Statistics

Data are presented as means ± standard deviations (SD). Student’s t-test was used for comparison of paired observations.

## Results

### Relationship between 15-LOX-1 Expression and Trimethylation of Histone H3-K4 at the 15-LOX-1 Promoter in HL-derived Cell Lines

**Figure 1:**
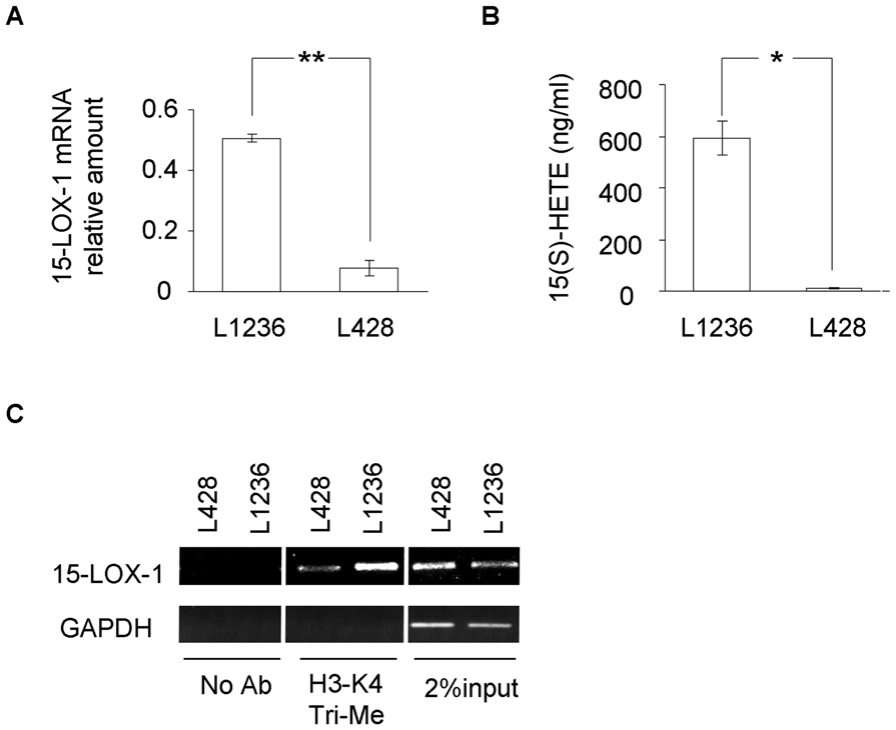
Association of 15-LOX-1 expression and activity with H3-K4 methylation status in HL-derived cell lines. (A) 15-LOX-1 mRNA levels in L1236 and L428 cells were measured using real-time PCR (n = 6). The expression levels were calculated relative to that of a calibrator sample (A549 cells ectopically expressing 15-LOX-1), levels of transcripts were expressed as the ratio vs. human β2 microglobulin. Bar, SD; ** p<0.01. (B) 15-HETE was measured as an indication of 15-LOX-1 activity in L1236 and L428 cells (n = 4). Cells were harvested after incubation with exogenous arachidonic acid (40 mM) at 37°C for 5 min. Bar, SD; * p<0.05. (C) ChIP assay for H3-K4 trimethylation at the 15-LOX-1 core promoter in L1236 and L428 cells. Primers B (see Materials and Methods and Fig. 3 A) were used for PCR amplification. Shown is one of four independent experiments.

In order to study the relation between 15-LOX-1 expression and chromatin remodelling status, 15-LOX-1 mRNA expression in the HL-derived L1236 and L428 cell lines was first examined. Consistent with our previous findings, real-time PCR showed that 15-LOX-1 transcription was strong in L1236 cells while very low in L428 cells (Fig. 1A). As shown in Fig. 1B, 15-LOX-1 catalytic activity in terms of 15-HETE formation upon challenge with exogenous arachidonic acid was strong in L1236 cells but nearly undetectable in L428 cells.

Histone H3 acetylation is positively correlated with 15-LOX-1 gene expression in cultured HL cells [17]; different lines of experimental evidence suggest that H3-K4 trimethylation is mostly associated with active gene expression [32]. Therefore, we asked whether H3-K4 methylation in the 15-LOX-1 promoter is related to transactivation of the enzyme. Using ChIP assay, strong H3-K4 trimethylation in the 15-LOX-1 promoter region of L1236 cells was observed. In contrast, this region was markedly less methylated in L428 cells (Fig. 1C). The results suggest an association of 15-LOX-1 promoter histone methylation status with transcriptional status of the gene in cultured HL cells.

### Histone Methyltransferase SMYD3 and Histone Demethylase SMCX Regulate 15-LOX-1 Gene Expression

**Figure 2:**
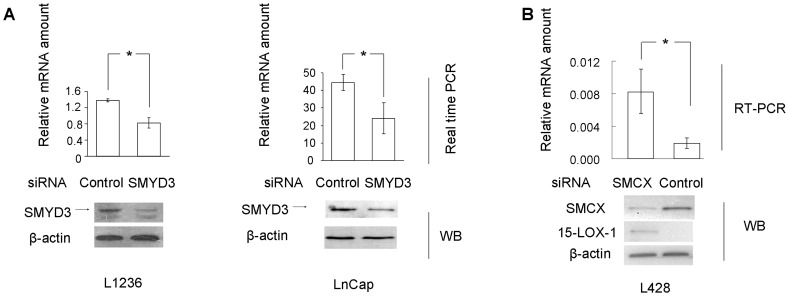
SMYD3 and SMCX regulate 15-LOX-1 expression in cultured HL-derived cells cells and prostate cancer cells. (A) Real-time PCR assay of 15-LOX-1 mRNA expression in L1236 and LNCaP cells treated with SMYD3 siRNAs or control siRNA (n = 4). The results were normalized to the mRNA level of beta-2 microglobulin. The efficiency of SMYD3 siRNA knocking down was evaluated using Western blots. Bar, SD; * p<0.05. (B) Real-time PCR assay and Western blot analysis of 15-LOX-1 mRNA and protein expression in L428 cells treated with SMCX siRNAs or control siRNA (n = 4). The real-time PCR data were normalized to the mRNA level of beta-2 microglobulin. The efficiency of SMCX siRNA knocking down was evaluated using Western blot and β-actin served as a loading control. Bar, SD; * p<0.05.

**Figure 3:**
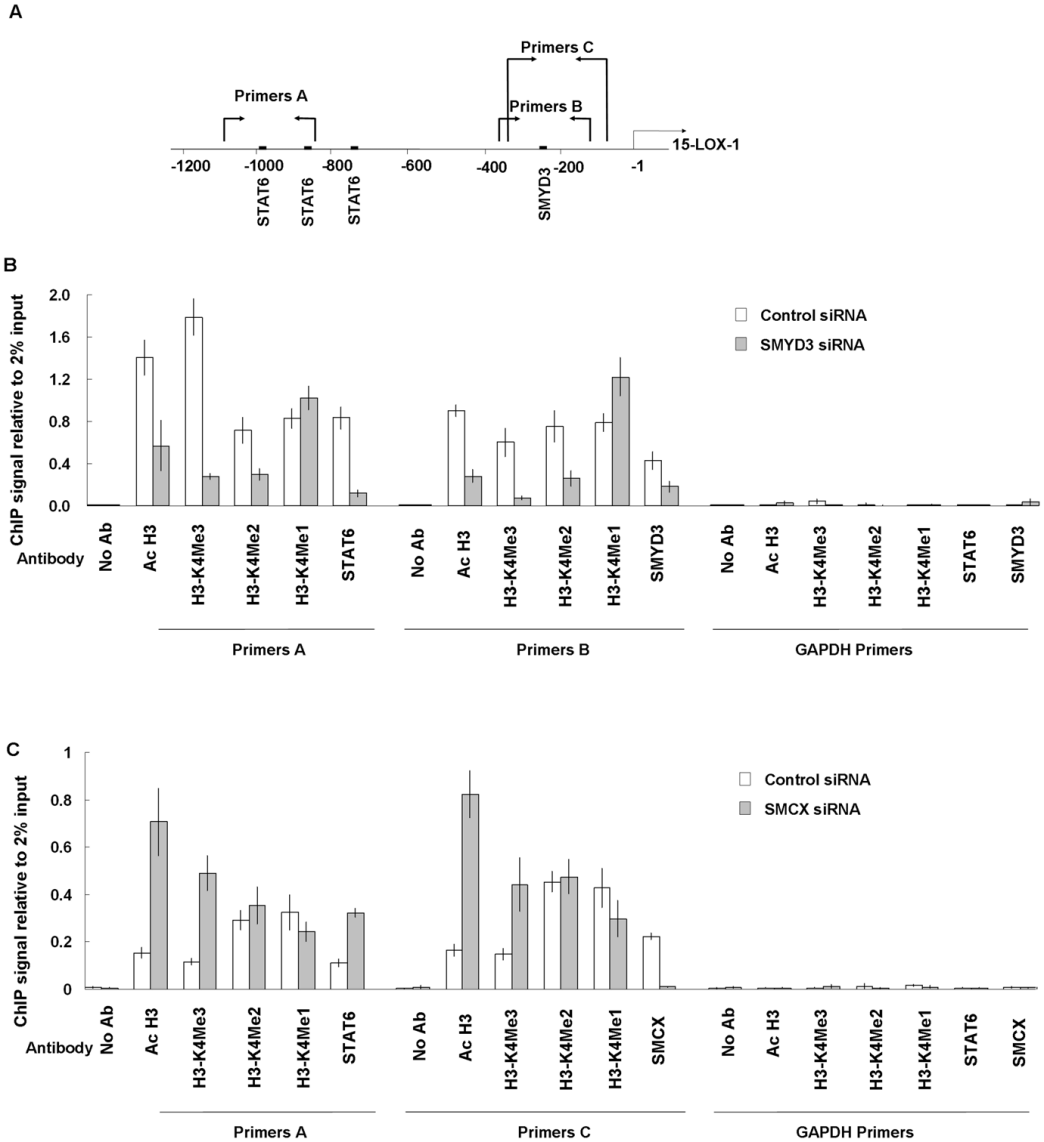
Modulation of the H3-K4 methylation/demethylation balance influences on 15-LOX-1 expression by affecting H3 acetylation and STAT6 occupancy at the 15-LOX-1 promoter. (A) Schematic presentation of the 15-LOX-1 promoter and PCR primer locations (relative to ATG) for the ChIP assay in relation to the three potential STAT6 binding motifs and SMYD3 binding site in the 15-LOX-1 promoter region. (B) Quantative ChIP assay for H3-K4 tri−/di−/monomethylation, acetylation, STAT6 and SMYD3 occupancy at the 15-LOX-1 promoter in L1236 cells treated with the SMYD3 siRNA or control siRNA. (C) Quantative ChIP assay for H3-K4 tri−/di−/monomethylation, acetylation, and STAT6 occupancy at the 15-LOX-1 promoter in L428 cells treated with the SMCX siRNA or control. Omission of antibodies (No Ab) was included in the whole experimental procedure, together with the PCR amplification of unrelated GAPDH gene, as appropriate controls. Data shown are from four independent experiments. Mean value of ChIP signals are normalized to 2% input. Input control is from non-immunoprecipitated total genomic DNA. Bar, SD.

Because 15-LOX-1 expression is closely related to H3-K4 methylation status, we next determined whether H3-K4 trimethylation is required for 15-LOX-1 transcription. Inhibition of SMYD3, a histone H3-K4–specific dimethyltransferase and trimethyltransferase, could lead to a hypo-methylated status of H3-K4 at the promoter region of its target genes [24], [33]. As shown in Fig. 2A and Fig. 3 A, B, knocking down SMYD3 expression using siRNA significantly inhibits H3-K4 methylation at the 15-LOX-1 promoter region and reduces 15-LOX-1 mRNA abundance in L1236. Expression of SMYD3 protein is undetectable in L428 cells (data not shown). To further test the hypothesis that 15-LOX-1 expression is controlled by the histone methylation status, the SMCX gene, coding a H3-K4 demethylase [34], was silenced in L428 cells by means of siRNA. The analyses showed that H3-K4 turned hyper-methylated at the 15-LOX-1 promoter and that the expression of the enzyme was induced at both mRNA and protein levels without IL-4 stimulation (Fig. 2B and Fig. 3C). The efficient silencing of SMYD3 or SMCX expression was verified by both reverse transcription-PCR (data not shown) and Western blot analyses (Fig. 2, lower panel).

Since 15-LOX-1 upregulation is suggested to be involved in the tumorigenesis of prostate cancer, it was also examined whether SMYD3 is required for 15-LOX-1 transcription in the prostate cancer cell line LNCaP. Strong SMYD3 protein expression was detected in this cell line and its inhibition by siRNA significantly repressed the 15-LOX-1 mRNA expression (Fig. 2A). Collectively, these data strongly suggest that 15-LOX-1 transcription activity could be controlled by histone methylation/demethylation.

### SMYD3 Regulates 15-LOX-1 Promoter Activity

**Figure 4:**
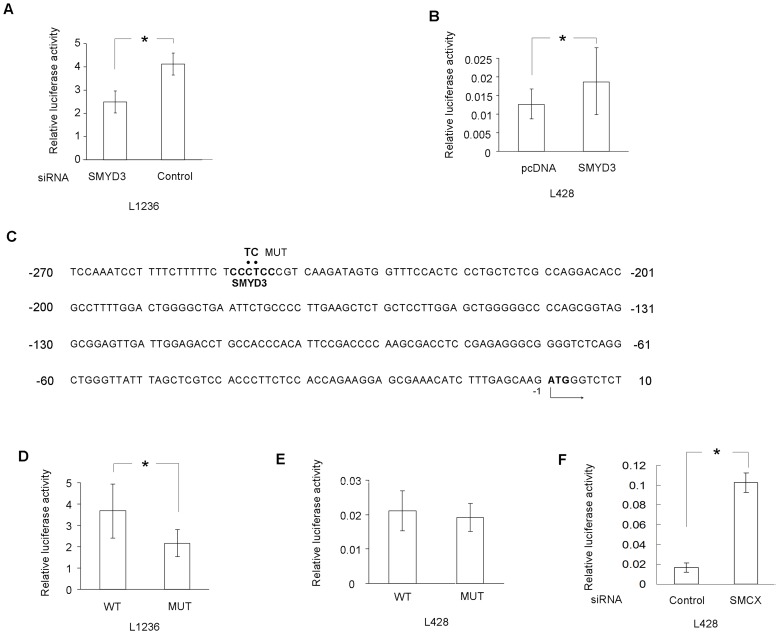
SMYD3 and SMCX regulates 15-LOX-1 expression at the transcriptional level. (A) SMYD3 depletion is associated with decreased 15-LOX-1 promoter activity. SMYD3 siRNA or control siRNA were contransfected with wild type (WT) pGL3-15-LOX-1 reporter plasmid into L1236 cells (n = 4). Variation in transfection efficiency was normalized by thymidine kinase-driven Renilla luciferase activity. Bar, SD; * p<0.05. (B) 15-LOX-1 transcription is induced by SMYD3 ectopic expression. SMYD3 expression vectors pcDNA-SMYD3 or empty vector pcDNA were cotransfected with WT pGL3-15-LOX-1 reporter plasmid into L428 cells (n = 4). Bar, SD; * p<0.05. (C) Sequence of the 15-LOX-1 core promoter region. A putative SMYD3 binding site is underlined. The sequence that was mutated in the transcriptional activity analysis of *cis*-acting elements is indicated by dots and substitutions are given above. −1 indicates the first nucleotide upstream of the transcription start site; the arrow indicates the first nucleotide of the first exon. (D and E) Mutation of the SMYD3 binding motif at the 15-LOX-1 promoter attenuates transcriptional activity in 15-LOX-1 positive cells. WT pGL3-15-LOX-1 (WT) or SMYD3 motif mutant reporter (MUT) were transfected into L1236 or L428 cells (n = 4). Bar, SD; * p<0.05. (F) SMCX knockdown leads to enhanced 15-LOX-1 promoter activity. SMCX siRNA or control siRNA were contransfected with wild type (WT) pGL3-15-LOX-1 reporter plasmid into L428 cells (n = 4). Bar, SD; * p<0.05.

To examine whether SMYD3 regulates 15-LOX-1 expression at the transcriptional level, we studied the effect of SMYD3 inhibition by siRNA on 15-LOX-1 promoter activity. A 15-LOX-1 promoter reporter plasmid named pGL3-15-LOX-1-WT (wild type) was developed in which a luciferase gene is driven by a 1081 bp fragment from the 15-LOX-1 promoter. L1236 cells were co-transfected with the pGL3-15-LOX-1-WT reporter plasmid and SMYD3 siRNA or unspecific control siRNA. As shown in Fig. 4A, after three days of cotransfection, SMYD3 inhibition was associated with a significant reduction of 15-LOX-1 transcription activity. These data suggest that SMYD3 is required for full 15-LOX-1 promoter activity in L1236 cells. To further investigate the regulatory function of SMYD3 in 15-LOX-1 transcription, L428 cells were cotransfected with pGL3-15-LOX-1-WT and a SMYD3 expression plasmid or mock vector (PC). Consistent with the results obtained in L1236 cells, overexpression of SMYD3 in L428 cells resulted in a significant up-regulation of 15-LOX-1 promoter activity three days post-transfection (Fig. 4B). Taken together, these observations indicate that SMYD3 regulates 15-LOX-1 expression at the transcriptional level.

As a transcription factor containing histone methyltransferase activity, SMYD3 directly binds to its potential target motif CCCTCC of downstream genes [24]. As shown in Fig. 4C, a potential SMYD3 binding motif lies in the core promoter region of 15-LOX-1 [35]. To determine if this motif is the direct target of SMYD3, a substitution mutant reporter vector was constructed by site mutagenesis. As shown in Fig. 4D, a decreased transcriptional activity was noted with the mutant reporter in L1236 cells, suggesting that the potential SMYD3 binding motif is a *cis*-acting element of 15-LOX-1 expression. Similar experiments were performed in L428 cells, but here a significant reduction in transcriptional activity was lacking when cells were transfected with the SMYD3 binding motif mutant reporter plasmid (Fig. 4E), probably because of the low SMYD3 expression in this cell line (data not shown).

### SMYD3 is Physically Associated with the 15-LOX-1 Promoter

Since the SMYD3 binding motif was shown to be involved in 15-LOX-1 transcription and SMYD3 regulates 15-LOX-1 promoter activity, we asked whether SMYD3 binds to the 15-LOX-1 promoter region *in vivo* in L1236 cells. To this end, ChIP assay was applied with primers encompassing the SMYD3 binding motif (Fig. 3A). We found that the 15-LOX-1 core promoter region covering the SMYD3 binding motif is occupied by SMYD3 in vivo and that the association was inhibited when SMYD3 was knocked down by siRNA (Fig. 3B). The specificity of the association was verified by the absence of specific sequence amplifications when antibodies were omitted and when primers for the unrelated GAPDH gene were applied in the PCR reaction.

### SMYD3 Inhibition Leads to Chromatin Remodelling and Reduced STAT6 Occupation at the 15-LOX-1 Promoter in L1236 Cells

Since SMYD3 exerts its transcription-activating effect by trimethylating H3-K4 at the promoter of target genes, we asked if SMYD3 contributes to 15-LOX-1 gene expression by altering histone modification and thereby transcription factor occupation. SMYD3 expression in L1236 cells was knocked down using siRNA and thereafter alterations in H3-K4 mono−/di−/trimethylation at the 15-LOX-1 promoter was examined by ChIP assay. As shown in Fig. 3 B, SMYD3 inhibition leads to decrease H3-K4 di- and trimethylation but not monomethylation at the promoter region of 15-LOX-1, indicating that SMYD3 is required for di- or trimethylation of H3-K4 at the 15-LOX-1 promoter.

Promoter H3-K4 di- or tri-methylation provide docking sites for certain protein complexes containing histone acetyltransferase (HAT) activity that in turn leads to increased accessibility for transcriptional activators [32]. We therefore investigated whether abolished H3-K4 di−/trimethylation impedes the 15-LOX-1 promoter occupancy of the transcription factor STAT6, a predominant *trans*-activator of the gene. We found that after three days of SMYD3 siRNA treatment, histone acetylation was diminished and the STAT6 binding was noticeably reduced at the 15-LOX-1 promoter (Fig. 3 B). Thus, data suggest that SMYD3 is required for H3-K4 di−/trimethylation of the 15-LOX-1 promoter in L1236 cells, promoting STAT6 access.

### SMCX Inhibition Affects Histone Modifications and Enhances STAT6 Binding at the 15-LOX-1 Promoter in L428 Cells

Because inhibition of H3-K4 demethylase upregulates 15-LOX-1 expression in L428 cells (Fig. 2 B), we sought to delineate the underlying mechanism. To this end, L428 cells were co-transfected with the pGL3-15-LOX-1-WT reporter plasmid and SMCX siRNA or control siRNA. As shown in Fig. 3 F, after three days of cotransfection, SMCX depletion led to a significant increase of 15-LOX-1 transcriptional activity. To further investigate the regulatory function of SMCX in 15-LOX-1 transcription, ChIP assay was applied. After three days of SMCX siRNA treatment, significant enhanced H3-K4 trimethylation but not di- or monomethylation of the 15-LOX-1 promoter region was detected in the L428 cells (Fig. 3 C). Consistent with the results presented in Fig. 2 B, it was also noted that inhibition of the H3-K4 demethylase with SMCX siRNA leads to a clear up-regulation of histone acetylation and STAT6 occupation without IL-4 treatment (Fig. 3C). These observations suggest that H3-K4 demethylase is required to keep the 15-LOX-1 promoter silenced in L428 cells by controlling chromatin folding and the accessibility of STAT6.

## Discussion

Chromatin remodelling including DNA and histone modification has an enormous potential for organizing and controlling information encoded by the genome. The genomic histone methylation/demethylation regulation mediated by the dynamic balance of HMTs/HDMs is a common event which is involved in as diverse cellular biological processes as gene transcription, X-chromosome inactivation, DNA damage repair, telomere function and DNA recombination [36]. H3-K4 methylation has been considered as a positive histone modification for transcription; it increases as gene expression becomes active [22]. H3-K4 hypermethylation is predominantly localized to the promoter region of genes and different lines of evidence suggest that disrupting HMTs or HDMs can modulate gene expression by changing the pattern of histone methylation at the promoter region [24], [34]. Because the 15-LOX-1 gene is highly regulated and specifically expressed only in certain types of human cells, we attempted to investigate a potential relationship between histone methylation and 15-LOX-1 expression.

The major finding in the present study is that histone H3-K4 methylation/demethylation regulates 15-LOX-1 expression in cultured HL and prostate cancer cells. To our knowledge, this is the first study showing genomic histone H3-K4 methylation/demethylation being involved in the regulation of expression of lipoxygenases. The evidence supporting a direct contribution of H3-K4 methylation/demethylation in 15-LOX-1 transcriptional regulation in cultured HL cells include the following observations: (1) The H3-K4 methylation status at the 15-LOX-1 promoter is associated with 15-LOX-1 mRNA and protein expression level; (2) introduction of HMT into the cells enhances 15-LOX-1 promoter activity; (3) inhibition of HMT SMYD3 represses 15-LOX-1 promoter activity and mRNA expression; (4) inhibition of HDM SMCX induces 15-LOX-1 gene transcription and protein expression. Thus, H3-K4 methylation/demethylation plays a significant role in regulating 15-LOX-1 expression, presumably contributing to the cell-specific profile of 15-LOX-1 expression in human cells. Specifically, these new findings depict the importance of histone methylation/demethylation regulation in the metabolism of eicosanoids in human cells.

**Figure 5:**
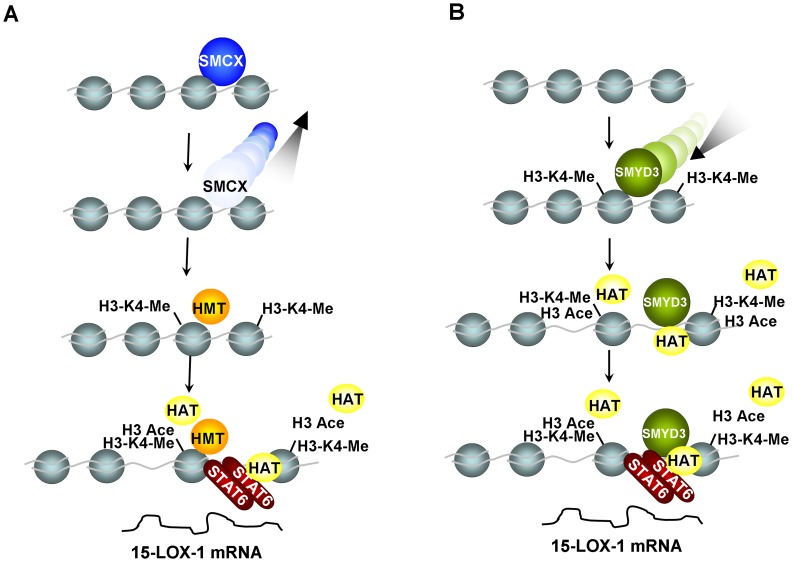
A model for HMT-mediated 15-LOX-1 transcriptional activation and HDM-mediated gene silencing through chromatin remodelling. In the 15-LOX-1 negative cell line L428, the 15-LOX-1 promoter region is occupied by HDM SMCX. Because H3-K4 is hypomethylated and H3 is hypoacetylated, the 15-LOX-1 promoter is not accessible to the transcriptional activator STAT6, and the gene transcription is repressed. Inhibition of SMCX with siRNA results in H3-K4 hypermethylation and subsequent H3 hyperacetylation through the recruitment of transcription complexes containing HAT activity, leading to an accessible promoter for STAT6. Promoter-bound STAT6 then recruits more HATs that in turn catalyze more H3 acetylation. These sequential events lead to transcriptional activation of the 15-LOX-1 gene (A). In L1236 cells with abundant 15-LOX-1 expression, the binding of SMYD3 to its motif in the 15-LOX-1 promoter region results in H3-K4 hypermethylation and 15-LOX-1 activation via a similar mechanism (B).

We further identified an HMT and an HDM which are involved in the H3-K4 methylation/demethylation of the 15-LOX-1 promoter and subsequent activation/repression of the transcription, respectively. Our study suggests that 15-LOX-1 is a direct target for the HMT SMYD3, based on the following evidence: (1) SMYD3 inhibition using siRNA represses 15-LOX-1 mRNA expression in L1236 and LNCaP cells; (2) SMYD3 siRNA inhibits 15-LOX-1 promoter activity in L1236 cells; (3) SMYD3 over-expression in L428 cells induces 15-LOX-1 promoter activity; (4) disruption of the potential SMYD3 binding motif using substitution mutation methodology reduces 15-LOX-1 promoter activity in L1236 cells. Furthermore, we found impaired occupancy of the transcription factor STAT6, a well-established IL-4/13 messenger and transcription activator of 15-LOX-1, at the promoter and diminished histone H3 acetylation following SMYD3 silencing and subsequent inhibition of H3-K4 di- and trimethylation in L1236 cells (Fig. 5A). It has been shown that SMYD3 can also directly interact with the ligand-binding domain of the estrogen receptor (ER) and be recruited to the proximal promoter regions of ER target genes upon gene induction [37]. The possibility of a direct physical interaction of SMYD3 with transcription factors involved in 15-LOX-1 transcriptional control, including STAT6, should be addressed in further studies.

Histone methylation can contribute to transcriptional regulation by recruiting transcription complexes which contain HAT activity. For example, the chromodomain of the chromatin remodelling protein Chd1 binds to methylated H3-K4, recruiting the yeast SAGA (Spt-Ada-Gcn5 HAT) complex which contains the H3 HAT GCN5 [38]. In the context of the present study, by governing histone acetylation status and the accessibility of STAT6 to the 15-LOX-1 promoter, SMYD3-mediated H3-K4 methylation may function as a critical element licensing 15-LOX-1 gene expression. Several lines of evidence suggest that SMYD3 is frequently overexpressed in human colorectal, liver and breast cancers, and its enhanced expression is considered essential for the growth of certain cancer cells [39]. In the present study, we found high expression of SMYD3 in the prostate cancer cell line LNCaP and weaker expression in prostate cancer PC3 cells. Since 15-LOX-1 has been suggested to play an important role in tumorigenesis and metastasis of prostate cancer [40], further investigation of the relation between SMYD3 expression and 15-LOX-1 transcriptional activation in prostate cancer *in vitro* and *in vivo* should be informative. Preliminary results show that SMYD3 is highly expressed in prostate cancer tissues and plays an important role for the growth and survival of prostate cancer cells (manuscript in preparation).

SMCX is a JmjC-domain-containing protein which possesses H3-K4 demethylase activity with a substrate preference for H3-K4-me2 and H3-K4-me3 and functions as a transcriptional repressor [34], [41]. Here, we describe that SMCX inhibition using siRNA activates 15-LOX-1 expression in L428 cells even without IL-4 stimulation. Further study based on ChIP assay suggested that SMCX exerts the 15-LOX-1 transcriptional repression effect by repressing H3-K4 trimethylation and H3 acetylation and consequently abolish the accessibility of STAT6 to its cognate binding motifs at the 15-LOX-1 promoter. We also depicted that SMCX binds within or very close to the 15-LOX-1core promoter region, although the specific binding sequence and binding site were not identified. Furthermore, our data suggest that SMCX represses 15-LOX-1 transcriptional activation through inhibiting H3-K4 trimethylation by its H3-K4 tri-demethylase activity (Fig. 5 A). However, as it has been reported that an SMCX complex isolated from HeLa cells contains additional chromatin modifiers, the histone deacetylases HDAC1 and HDAC2 [34], it is possible that SMCX can mediate transcription repression also independently of its demethylase activity.

In the present study, a reduction of 15-LOX-1 protein two days after SMYD3 siRNA treatment was not observed. This, however, is not surprising considering the stability of the 15-LOX-1 protein in L1236 cells; neither 15-LOX-1 siRNA nor the translation inhibitor cycloheximide was able to knock down the 15-LOX-1 protein levels after two or three days treatment (data not shown).

Collectively, our data suggest that histone methylation/demethylation at the 15-LOX-1 promoter is important in the transcriptional regulation of the gene in cultured cells. Thus, the process of 15-LOX-1 related eicosanoid oxygenation is controlled also by the dynamic balance between HMTs and HDMs.

## References

[1] Schewe T (2002) 15-lipoxygenase-1: a prooxidant enzyme. Biol Chem 383: 365–374. 12033428 10.1515/BC.2002.041

[2] Kuhn H, O’Donnell VB (2006) Inflammation and immune regulation by 12/15-lipoxygenases. Prog Lipid Res 45: 334–356. 16678271 10.1016/j.plipres.2006.02.003

[3] Dobrian AD, Lieb DC, Cole BK, Taylor-Fishwick DA, Chakrabarti SK, et al (2011) Functional and pathological roles of the 12- and 15-lipoxygenases. Prog Lipid Res 50: 115–131. 20970452 10.1016/j.plipres.2010.10.005 PMC3012140

[4] Zuo X, Peng Z, Wu Y, Moussalli MJ, Yang XL, et al (2012) Effects of Gut-Targeted 15-LOX-1 Transgene Expression on Colonic Tumorigenesis in Mice. J Natl Cancer Inst 104: 709–716. 22472308 10.1093/jnci/djs187 PMC3341308

[5] Kelavkar UP, Hutzley J, McHugh K, Allen KG, Parwani A (2009) Prostate tumor growth can be modulated by dietarily targeting the 15-lipoxygenase-1 and cyclooxygenase-2 enzymes. Neoplasia 11: 692–699. 19568414 10.1593/neo.09334 PMC2697355

[6] Claesson HE (2009) On the biosynthesis and biological role of eoxins and 15-lipoxygenase-1 in airway inflammation and Hodgkin lymphoma. Prostaglandins Other Lipid Mediat 89: 120–125. 19130894 10.1016/j.prostaglandins.2008.12.003

[7] Zhao J, O’Donnell VB, Balzar S, St Croix CM, Trudeau JB, et al (2011) 15-Lipoxygenase 1 interacts with phosphatidylethanolamine-binding protein to regulate MAPK signaling in human airway epithelial cells. Proc Natl Acad Sci U S A 108: 14246–14251. 21831839 10.1073/pnas.1018075108 PMC3161579

[8] Rong S, Cao Q, Liu M, Seo J, Jia L, et al (2012) Macrophage 12/15 lipoxygenase expression increases plasma and hepatic lipid levels and exacerbates atherosclerosis. J Lipid Res 53: 686–695. 22279185 10.1194/jlr.M022723 PMC3307645

[9] Chen B, Tsui S, Boeglin WE, Douglas RS, Brash AR, et al (2006) Interleukin-4 induces 15-lipoxygenase-1 expression in human orbital fibroblasts from patients with Graves disease. Evidence for anatomic site-selective actions of Th2 cytokines. J Biol Chem 281: 18296–18306. 16675443 10.1074/jbc.M603484200

[10] Wu SH, Liao PY, Yin PL, Zhang YM, Dong L (2009) Elevated expressions of 15-lipoxygenase and lipoxin A4 in children with acute poststreptococcal glomerulonephritis. Am J Pathol 174: 115–122. 19095947 10.2353/ajpath.2009.080671 PMC2631324

[11] van Leyen K, Duvoisin RM, Engelhardt H, Wiedmann M (1998) A function for lipoxygenase in programmed organelle degradation. Nature 395: 392–395. 9759730 10.1038/26500

[12] Ezaki J, Kominami E, Ueno T (2011) Peroxisome degradation in mammals. IUBMB Life 63: 1001–1008. 21990012 10.1002/iub.537

[13] Zuo X, Morris JS, Shureiqi I (2008) Chromatin modification requirements for 15-lipoxygenase-1 transcriptional reactivation in colon cancer cells. J Biol Chem 283: 31341–31347. 18799463 10.1074/jbc.M803729200 PMC2581547

[14] Das S, Roth CP, Wasson LM, Vishwanatha JK (2007) Signal transducer and activator of transcription-6 (STAT6) is a constitutively expressed survival factor in human prostate cancer. Prostate 67: 1550–1564. 17705178 10.1002/pros.20640

[15] Conrad DJ, Lu M (2000) Regulation of human 12/15-lipoxygenase by Stat6-dependent transcription. Am J Respir Cell Mol Biol 22: 226–234. 10657944 10.1165/ajrcmb.22.2.3786

[16] Liu C, Xu D, Sjoberg J, Forsell P, Bjorkholm M, et al (2004) Transcriptional regulation of 15-lipoxygenase expression by promoter methylation. Exp Cell Res 297: 61–67. 15194425 10.1016/j.yexcr.2004.02.014

[17] Liu C, Schain F, Han H, Xu D, Andersson-Sand H, et al (2012) Epigenetic and transcriptional control of the 15-lipoxygenase-1 gene in a Hodgkin lymphoma cell line. Exp Cell Res 318: 169–176. 22094113 10.1016/j.yexcr.2011.10.017

[18] Shankaranarayanan P, Chaitidis P, Kuhn H, Nigam S (2001) Acetylation by histone acetyltransferase CREB-binding protein/p300 of STAT6 is required for transcriptional activation of the 15-lipoxygenase-1 gene. J Biol Chem 276: 42753–42760. 11509556 10.1074/jbc.M102626200

[19] Kamitani H, Taniura S, Ikawa H, Watanabe T, Kelavkar UP, et al (2001) Expression of 15-lipoxygenase-1 is regulated by histone acetylation in human colorectal carcinoma. Carcinogenesis 22: 187–191. 11159758 10.1093/carcin/22.1.187

[20] Kelavkar UP, Harya NS, Hutzley J, Bacich DJ, Monzon FA, et al (2007) DNA methylation paradigm shift: 15-lipoxygenase-1 upregulation in prostatic intraepithelial neoplasia and prostate cancer by atypical promoter hypermethylation. Prostaglandins Other Lipid Mediat 82: 185–197. 17164146 10.1016/j.prostaglandins.2006.05.015

[21] Zuo X, Shen L, Issa JP, Moy O, Morris JS, et al.. (2008) 15-Lipoxygenase-1 transcriptional silencing by DNA methyltransferase-1 independently of DNA methylation. Faseb J. 10.1096/fj.07-098301 PMC2410033 18198215

[22] Berger SL (2007) The complex language of chromatin regulation during transcription. Nature 447: 407–412. 17522673 10.1038/nature05915

[23] Ordovas JM, Smith CE (2010) Epigenetics and cardiovascular disease. Nat Rev Cardiol 7: 510–519. 20603647 10.1038/nrcardio.2010.104 PMC3075976

[24] Hamamoto R, Furukawa Y, Morita M, Iimura Y, Silva FP, et al (2004) SMYD3 encodes a histone methyltransferase involved in the proliferation of cancer cells. Nat Cell Biol 6: 731–740. 15235609 10.1038/ncb1151

[25] Cock-Rada AM, Medjkane S, Janski N, Yousfi N, Perichon M, et al (2012) SMYD3 promotes cancer invasion by epigenetic upregulation of the metalloproteinase MMP-9. Cancer Res 72: 810–820. 22194464 10.1158/0008-5472.CAN-11-1052 PMC3299564

[26] Niu X, Zhang T, Liao L, Zhou L, Lindner DJ, et al (2012) The von Hippel-Lindau tumor suppressor protein regulates gene expression and tumor growth through histone demethylase JARID1C. Oncogene 31: 776–786. 21725364 10.1038/onc.2011.266 PMC4238297

[27] Marafioti T, Hummel M, Foss HD, Laumen H, Korbjuhn P, et al (2000) Hodgkin and reed-sternberg cells represent an expansion of a single clone originating from a germinal center B-cell with functional immunoglobulin gene rearrangements but defective immunoglobulin transcription. Blood 95: 1443–1450. 10666223

[28] Xu D, Gruber A, Peterson C, Pisa P (1998) Telomerase activity and the expression of telomerase components in acute myelogenous leukaemia. Br J Haematol 102: 1367–1375. 9753073 10.1046/j.1365-2141.1998.00969.x

[29] Andersson E, Schain F, Svedling M, Claesson HE, Forsell PK (2006) Interaction of human 15-lipoxygenase-1 with phosphatidylinositol bisphosphates results in increased enzyme activity. Biochim Biophys Acta 1761: 1498–1505. 17052953 10.1016/j.bbalip.2006.09.007

[30] Wittwer J, Marti-Jaun J, Hersberger M (2006) Functional polymorphism in ALOX15 results in increased allele-specific transcription in macrophages through binding of the transcription factor SPI1. Hum Mutat 27: 78–87. 16320347 10.1002/humu.20273

[31] Ge Z, Liu C, Bjorkholm M, Gruber A, Xu D (2006) Mitogen-activated protein kinase cascade-mediated histone H3 phosphorylation is critical for telomerase reverse transcriptase expression/telomerase activation induced by proliferation. Mol Cell Biol 26: 230–237. 16354694 10.1128/MCB.26.1.230-237.2006 PMC1317632

[32] Eissenberg JC, Shilatifard A (2010) Histone H3 lysine 4 (H3K4) methylation in development and differentiation. Dev Biol 339: 240–249. 19703438 10.1016/j.ydbio.2009.08.017 PMC3711867

[33] Liu C, Fang X, Ge Z, Jalink M, Kyo S, et al (2007) The telomerase reverse transcriptase (hTERT) gene is a direct target of the histone methyltransferase SMYD3. Cancer Res 67: 2626–2631. 17363582 10.1158/0008-5472.CAN-06-4126

[34] Tahiliani M, Mei P, Fang R, Leonor T, Rutenberg M, et al (2007) The histone H3K4 demethylase SMCX links REST target genes to X-linked mental retardation. Nature 447: 601–605. 17468742 10.1038/nature05823

[35] Kelavkar U, Wang S, Montero A, Murtagh J, Shah K, et al (1998) Human 15-lipoxygenase gene promoter: analysis and identification of DNA binding sites for IL-13-induced regulatory factors in monocytes. Mol Biol Rep 25: 173–182. 9700053 10.1023/a:1006813009006

[36] Quina AS, Buschbeck M, Di Croce L (2006) Chromatin structure and epigenetics. Biochem Pharmacol 72: 1563–1569. 16836980 10.1016/j.bcp.2006.06.016

[37] Kim H, Heo K, Kim JH, Kim K, Choi J, et al (2009) Requirement of histone methyltransferase SMYD3 for estrogen receptor-mediated transcription. J Biol Chem 284: 19867–19877. 19509295 10.1074/jbc.M109.021485 PMC2740412

[38] Pray-Grant MG, Daniel JA, Schieltz D, Yates JR 3rd, Grant PA (2005) Chd1 chromodomain links histone H3 methylation with SAGA- and SLIK-dependent acetylation. Nature 433: 434–438. 15647753 10.1038/nature03242

[39] Silva FP, Hamamoto R, Kunizaki M, Tsuge M, Nakamura Y, et al.. (2007) Enhanced methyltransferase activity of SMYD3 by the cleavage of its N-terminal region in human cancer cells. Oncogene. 10.1038/sj.onc.1210929 17998933

[40] Kelavkar U, Lin Y, Landsittel D, Chandran U, Dhir R (2006) The yin and yang of 15-lipoxygenase-1 and delta-desaturases: dietary omega-6 linoleic acid metabolic pathway in prostate. J Carcinog 5: 9. 16566819 10.1186/1477-3163-5-9 PMC1440856

[41] Jensen LR, Amende M, Gurok U, Moser B, Gimmel V, et al (2005) Mutations in the JARID1C gene, which is involved in transcriptional regulation and chromatin remodeling, cause X-linked mental retardation. Am J Hum Genet 76: 227–236. 15586325 10.1086/427563 PMC1196368

